# Supplementary material for: Microbial consortia driving (ligno)cellulose transformation in agricultural woodchip bioreactors
Source: Appl Environ Microbiol. 2024 Nov 11;90(12):e01742-24. doi: 10.1128/aem.01742-24 (PMC11653725; doi:10.1128/aem.01742-24)
Supplement: Supplemental figures — Figures S1 to S8. [file aem.01742-24-s0001.docx]

# Supplementary figures

**Microbial consortia driving lignocellulose transformation in agricultural woodchip bioreactors**

Valerie C. Schiml, Juline M. Walter, Live H. Hagen, Aniko Varnai, Linda L. Bergaust, Arturo Vera Ponce De Leon, Lars Elsgaard, Lars R. Bakken, Magnus Ø. Arntzen

**Figure S1: Gas measurements of enrichment culture headspaces.**

**Figure S2: Read abundance of denitrification genes.**

**Figure S3: Complete N-metabolism.**

**Figure S4: Metagenomic and metaproteomics detection of enzymes for denitrification and DNRA.**

**Figure S5: Module completion fractions (mcf) for all MAGs and KEGG modules.**

**Figure S6: Expressed CAZymes.**

**Figure S7: Changes in diversity of microbial communities in WBR.**

**Figure S8: A schematic of the WBR design**


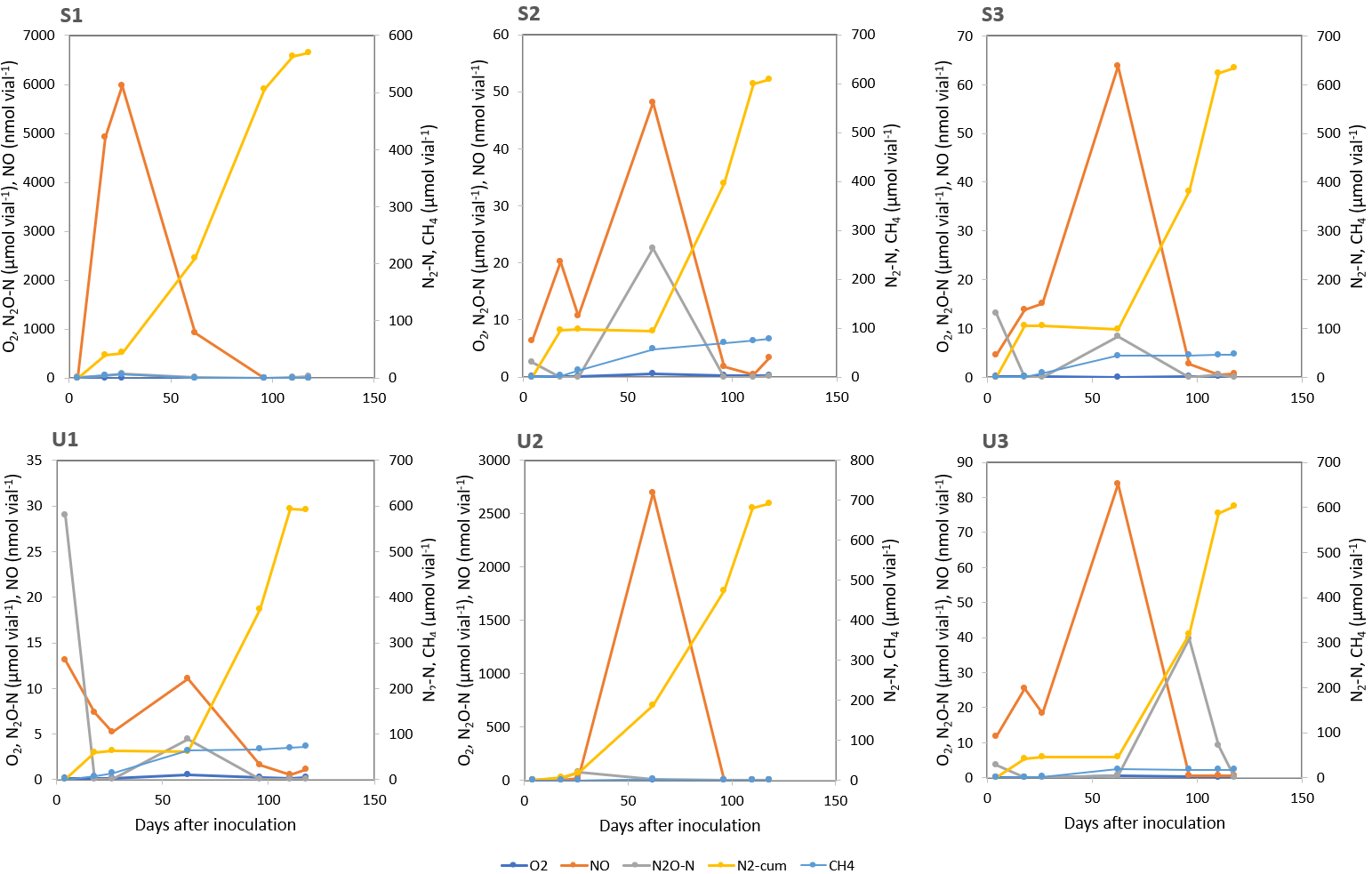


**Figure S1: Gas measurements of enrichment culture headspaces.** The figure shows the concentrations of O_2_ (µmol vial^-1^), NO (nmol vial^-1^), N_2_O-N (µmol vial^-1^), cumulative N_2_ (µmol vial^-1^), and methane (µmol vial^-1^) over time for the 2^nd^ /subculture enrichment shown in Figure 2. The concentration of available N was given as 5 mM KNO_3_ at the start of the incubation, and additional 5 mM KNO_3_ were added at 61 days and 103 days. O_2_ concentration was always ~0.1 µmol vial^-1^ except right after addition of more KNO_3_ which led to a transient increase to ~0.6 µmol vial^-1^ O_2_. Sample names indicate sampling from WBR surface (S) or under water (U), in three measurements (1-3).


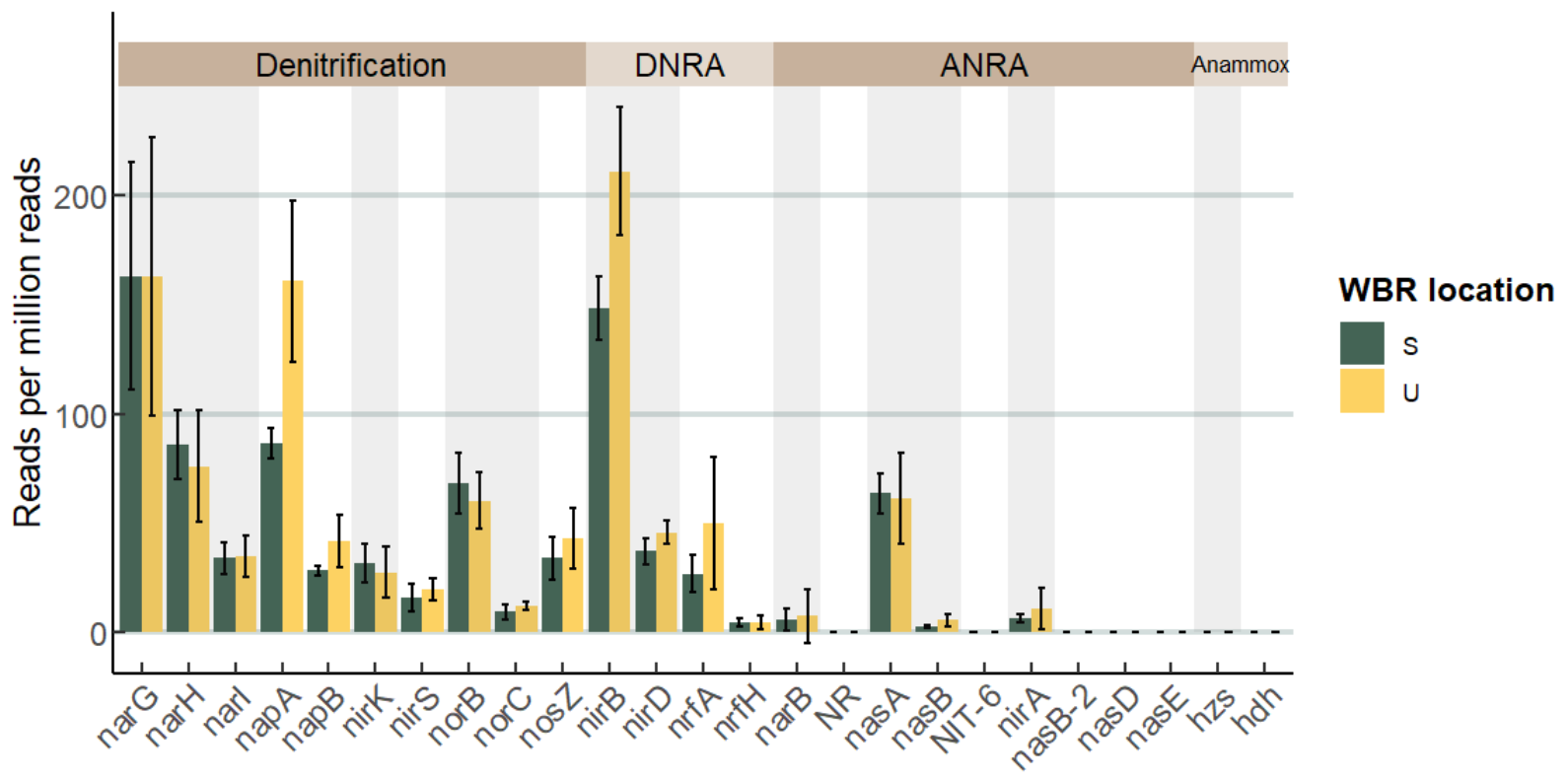


**Figure S2: Read abundance of genes involved in various parts of N metabolism.** The figure shows the read abundances of genes whose products are involved in denitrification (*narGHI, napAB, nirK, nirS, norBC, nosZ*), DNRA (*nirBD*, *nrfA*, *nrfH*), assimilatory nitrate reduction (ANRA; *narB, NR, nasAB, NIT-6, nirA, nasBDE*), and anammox (*hzs, hdh*). Values are given in reads-per-million at the surface (S; 15 cm depth) and under water (U; 60-80 cm depth) locations of the WBR for replicate measurements (n=3).


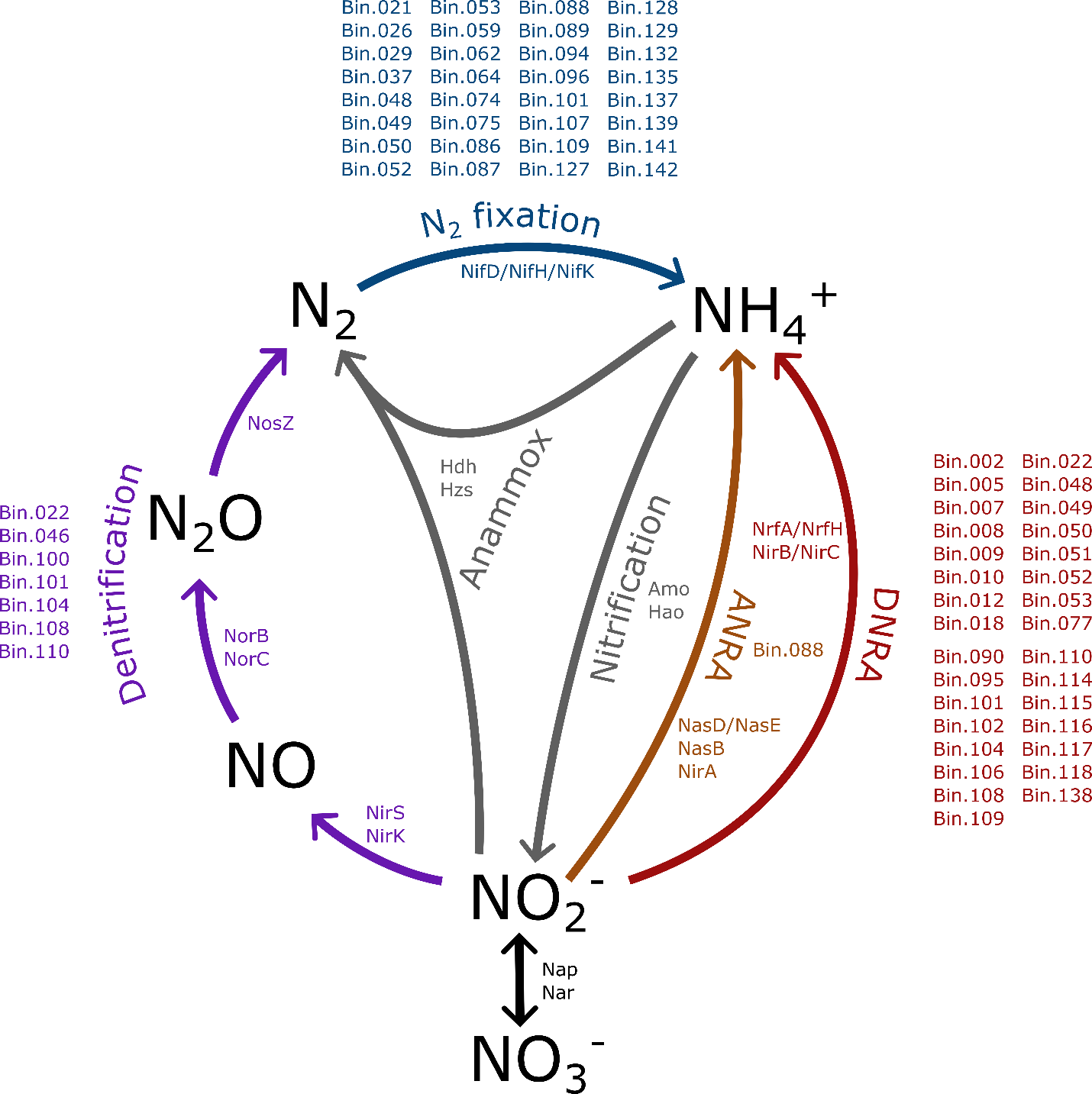


**Figure S3: Complete N-metabolism.** The figure shows a distribution of MAGs potentially involved in the various steps of N-metabolism. MAGs with a module completion fraction (mcf) of 1, i.e., all required enzymes for a given biochemical pathway are present, are listed. For nitrification and anammox, no MAGs could meet this criterium. Abbreviations: Dissimilatory nitrate reduction to ammonia (DNRA), Assimilatory nitrate reduction to ammonia (ANRA).


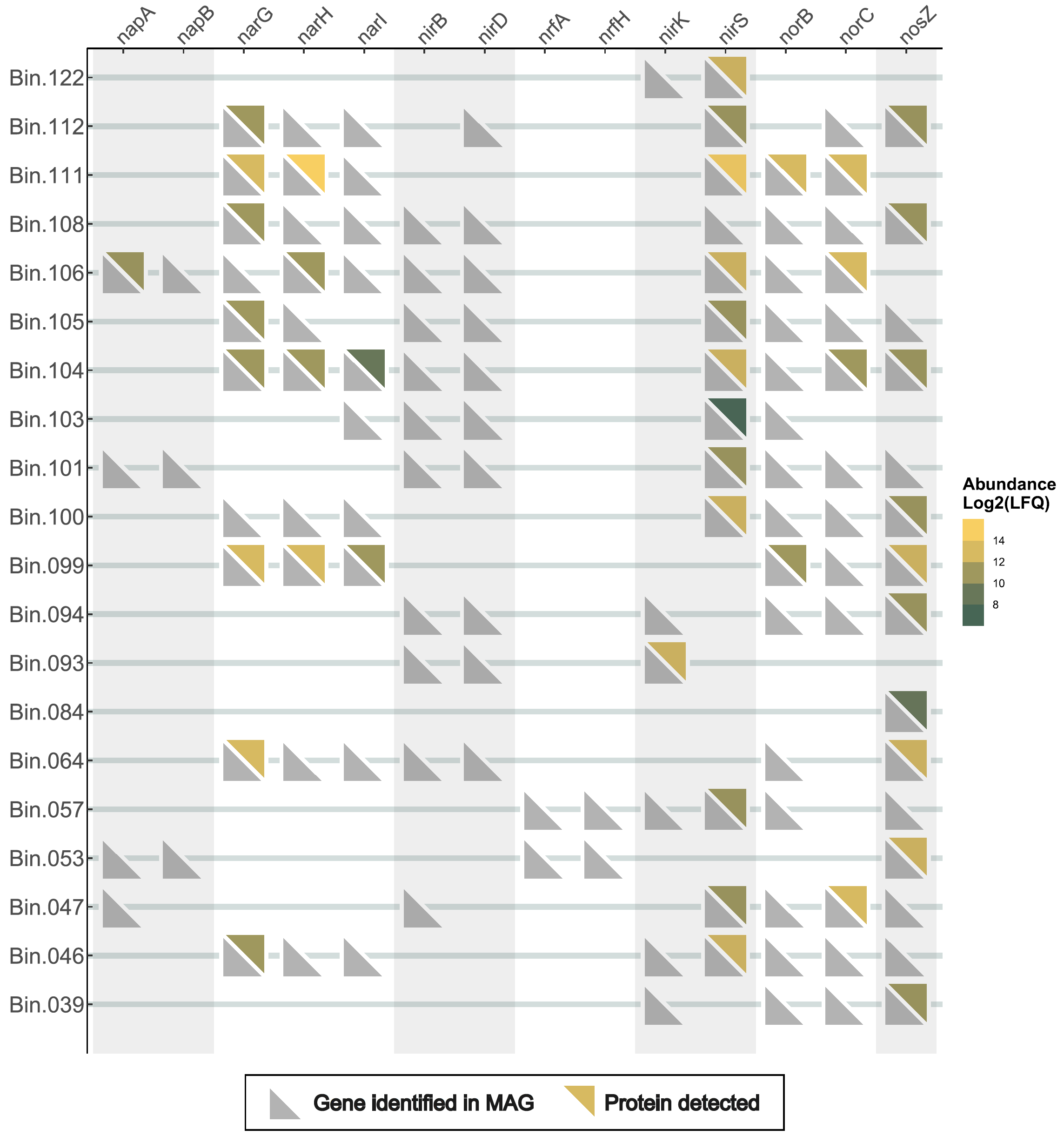


**Figure S4: Metagenomic and metaproteomics detection of enzymes for denitrification and DNRA.** The figure shows identified genes for denitrification and DNRA, and the corresponding protein abundance. Only MAGs with at least one complete enzyme and at least one enzyme expressed are shown. This list correspond to the MAGs listed in Figure 4.


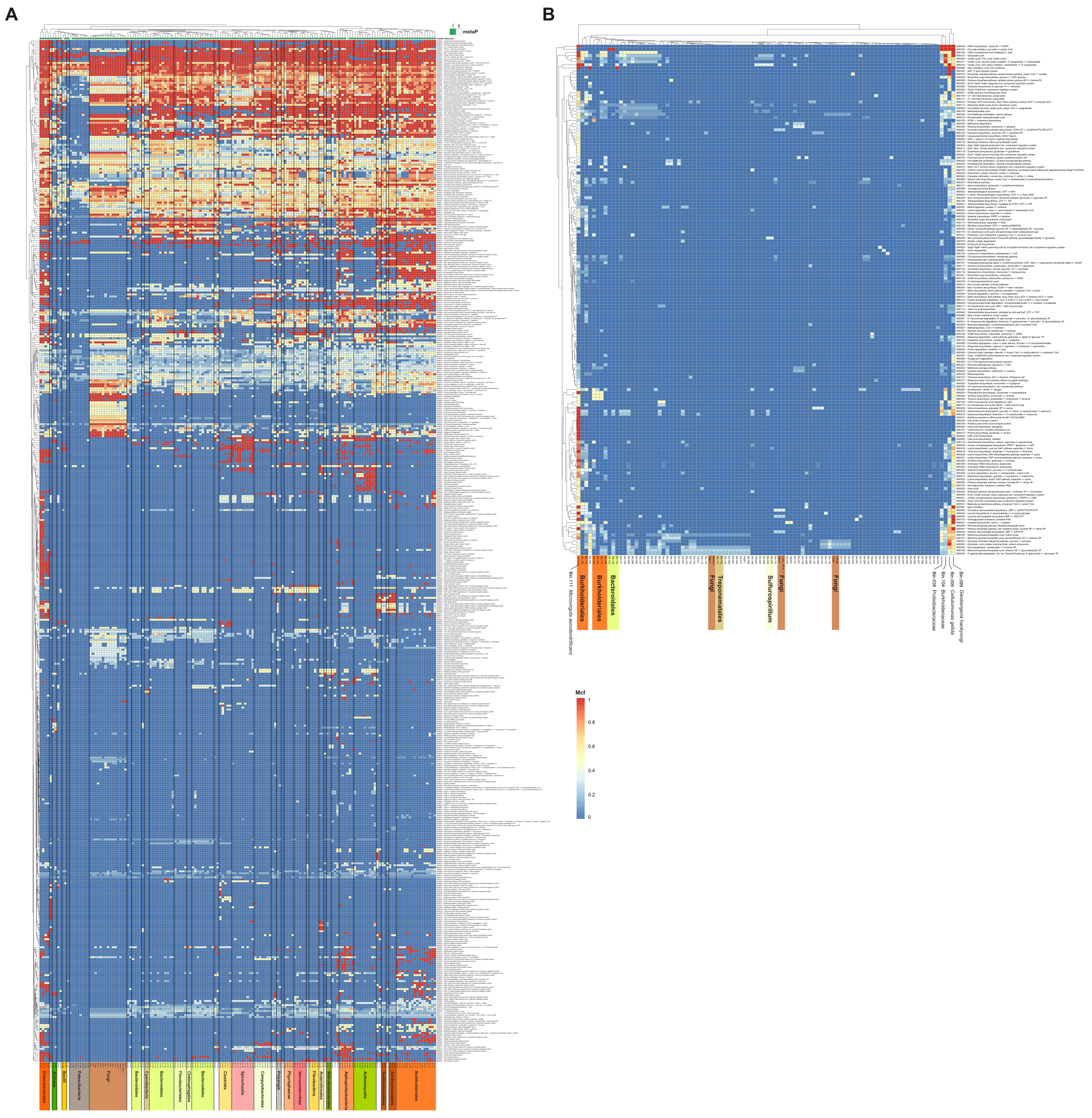


**Figure S5: Module completion fractions (mcf) for all MAGs and KEGG modules.** The figure shows heatmaps based on metagenomics (**A**) and metaproteomics (**B**) of all the MAGs (x-axis), KEGG modules (y-axis) and color as the module completion fraction (mcf) where mcf=1 (red) when all required enzymes for a given biochemical pathway are present in the MAG’s genome (for A) or expressed (for B). Taxonomical clusters are indicated underneath with colors. Only KEGG modules where at least one MAG had a mcf > 0 are shown.


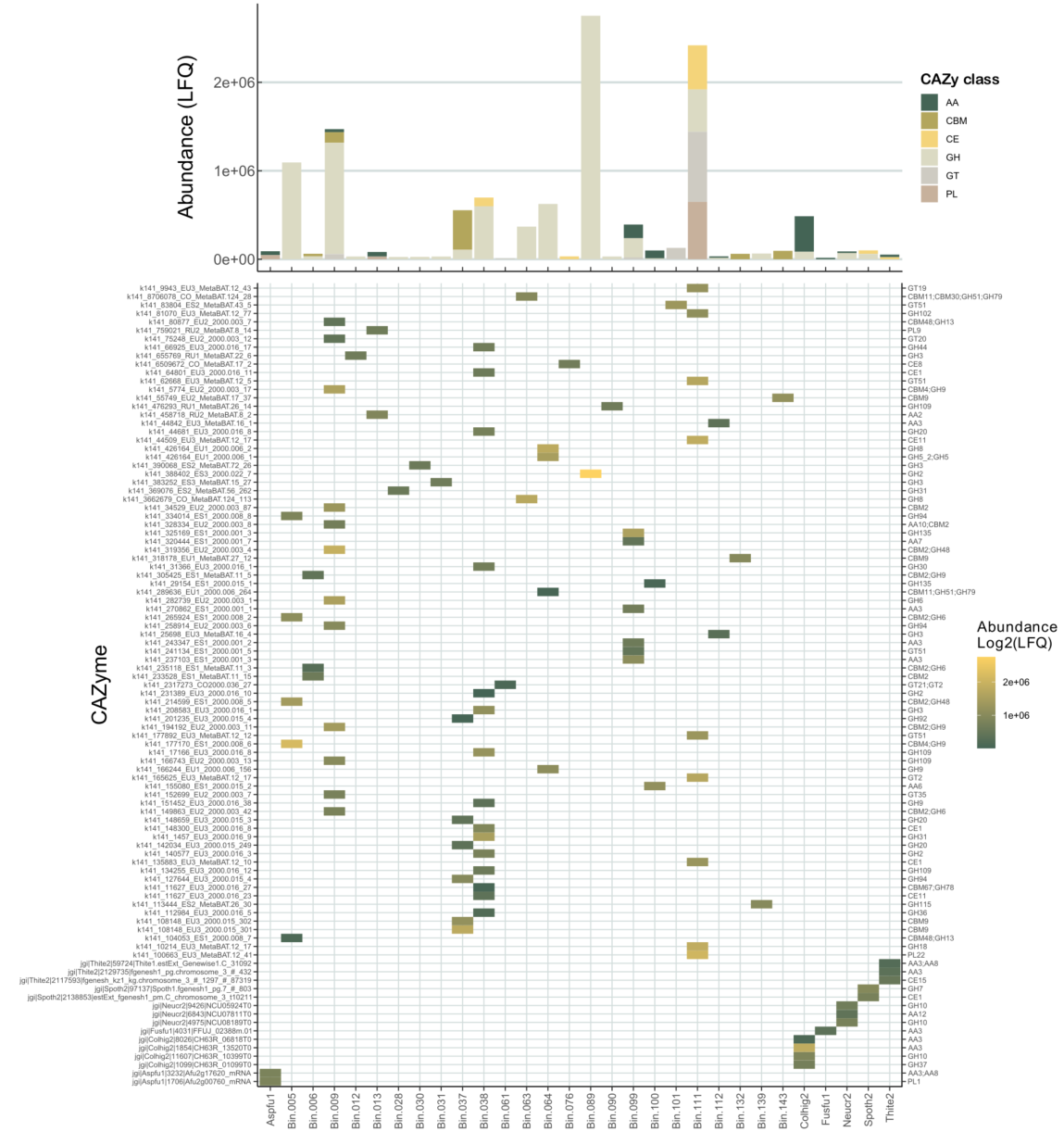


**Figure S6: Expressed CAZymes.** The figure shows a summarizing barplot (top) and heatmap (bottom) of all CAZymes expressed by the various MAGs. These are the same as depicted in Figure 4 but with abundances. In total, 24 MAGs and 6 fungi contribute to the lignocellulose degradation using glycoside hydrolases (GH), polysaccharide lyases (PL), carbohydrate esterases (CE), auxiliary activities (AA), glycosyl transferases (GT), and carbohydrate binding modules (CBM). Abbreviations: Aspfu1 - *Aspergillus fumigatus*, Colhig2 - *Colletotrichum higginsianum*, Fusfu1 - *Fusarium fujikuroi*, Neucr2 - *Neurospora crassa*, Spoth2 *- Thermothelomyces thermophilus*, Thite2- *Thermothielavioides terrestris.*


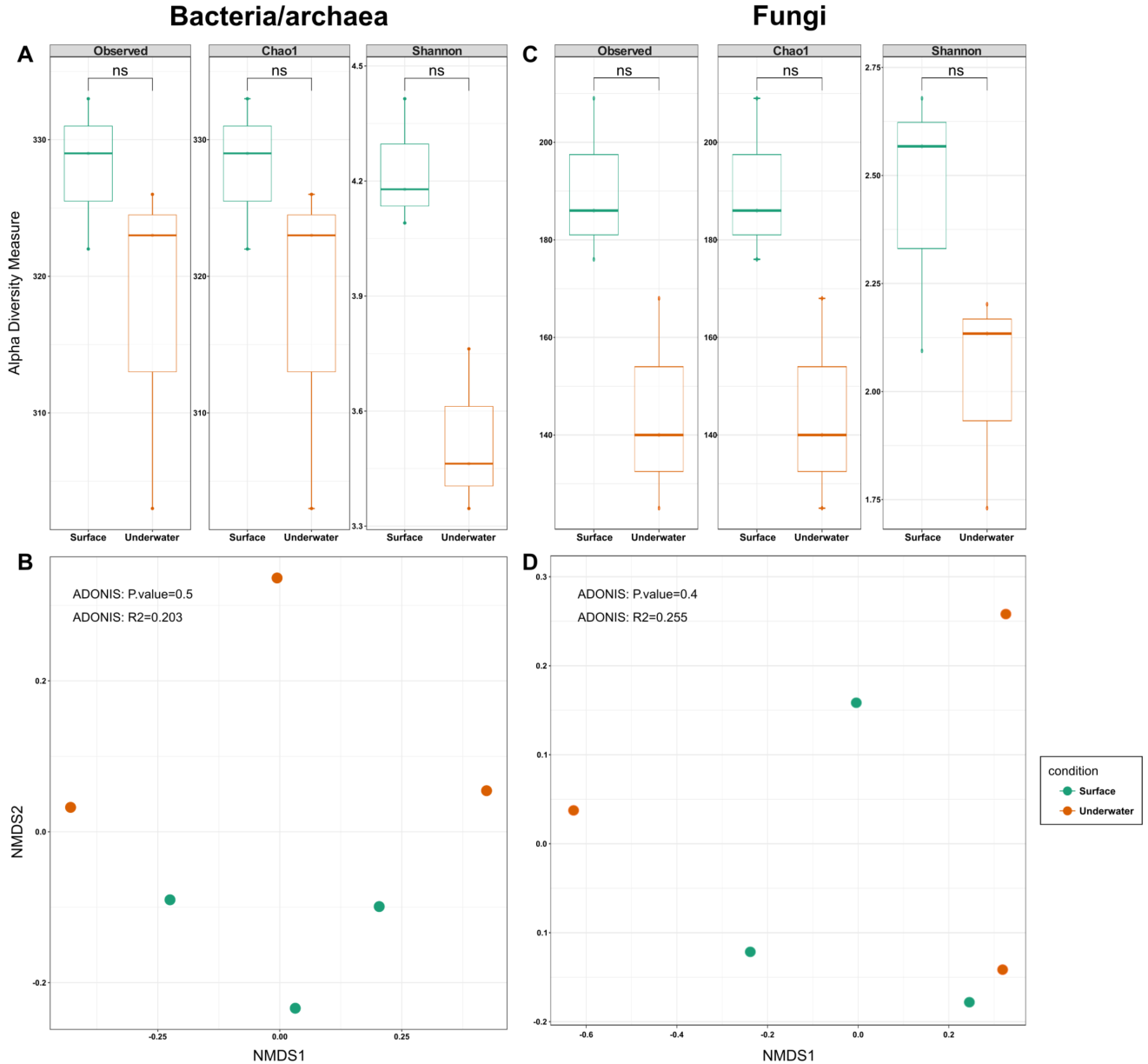


**Figure S7: Changes in diversity of microbial communities in WBR.** The figure shows a comparison between surface and underwater samples for bacterial ASVs (A-B), and fungal OTUs (C-D). A, C) Alpha-diversity in means of observed, Caho1 and Shannon indexes, B, D) NMDS plot showing cluster of samples by Bray-Curtis distance dissimilarity.


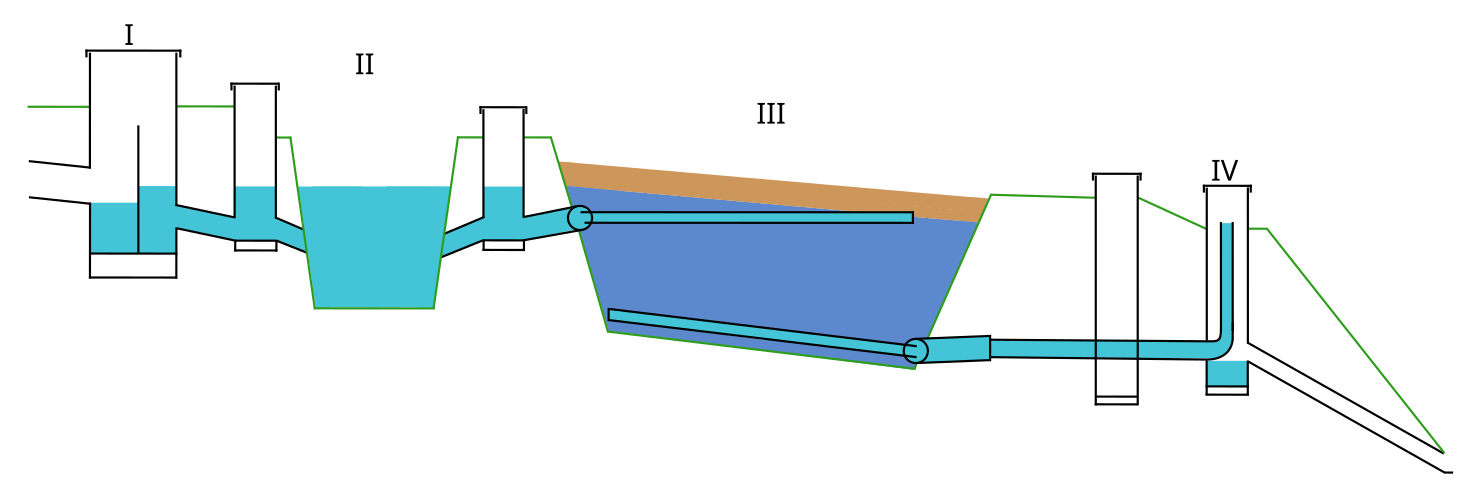


**Figure S8: A schematic of the WBR design.** The figure shows a horizontal schematic of the WBR in Dundelum, Haderslev, Denmark, used in this study. Chambers I, II, and IV are designed to regulate the WBR saturation level in response to changes in rain and flood. Chamber III depicts the vertical flow WBR where samples were taken at two depths, 15 cm (S; surface samples) and 60-80 cm (U; underwater samples). Figure is adapted from a technical drawing of the facility, found at <https://dcapub.au.dk/djfpublikation/djfpdf/DCArapport190.pdf>, Figure 25.
